# Typhoon disaster state information extraction for Chinese texts

**DOI:** 10.1038/s41598-024-58585-8

**Published:** 2024-04-04

**Authors:** Peng Ye, Chunju Zhang, Mingzhu Chen, Shengcai Li

**Affiliations:** 1 Urban Planning and Development Institute, Yangzhou University, Yangzhou, 225127 China; 2 College of Architectural Science and Engineering, Yangzhou University, Yangzhou, 225127 China; 3 School of Civil Engineering, Hefei University of Technology, Hefei, 230009 China; 4 Key Laboratory of Virtual Geographic Environment, Ministry of Education, Nanjing Normal University, Nanjing, 210023 China; 5 Jiangsu Center for Collaborative Innovation in Geographical Information Resource Development and Application, Nanjing, 210023 China

**Keywords:** Typhoon disaster, State information extraction, Spatio-temporal semantic unit, Typhoon disaster semantic vector, State information fusion, Climate sciences, Natural hazards, Engineering

## Abstract

Typhoon disasters undergo a complex evolutionary process influenced by temporal changes, and investigating this process constitutes the central focus of geographical research. As a key node within the typhoon disaster process, the state serves as the foundation for gauging the dynamics of the disaster. The majority of current approaches to disaster information extraction rely on event extraction methods to acquire fundamental elements, including disaster-causing factors, disaster-bearing bodies, disaster-pregnant environment and the extent of damage. Due to the dispersion of various disaster information and the diversity of time and space, it is a challenge for supporting the analysis of the typhoon disaster process. In this paper, a typhoon disaster state information extraction (TDSIE) method for Chinese texts is proposed, which aims to facilitate the systematic integration of fragmented typhoon disaster information. First, the integration of part-of-speech tagging with spatio-temporal information extraction is employed to achieve the tagging of typhoon disaster texts. Second, within the framework of spatio-temporal semantic units, the typhoon disaster semantic vector is constructed to facilitate the identification of information elements of typhoon disaster states. Third, co-referential state information fusion is performed based on spatio-temporal cues. Experimental analysis, conducted using online news as the data source, reveals that the TDSIE achieves precision and recall rates consistently surpassing 85%. The typhoon disaster state information derived from the TDSIE allows for the analysis of spatio-temporal patterns, evolutionary characteristics, and activity modes of typhoon disasters across various scales. Therefore, TDSIE serves as valuable support for investigating the inherent process properties of typhoon disasters.

## Introduction

Typhoons are one of the most destructive natural disasters in the world^1^. Over the past 40 years, the frequency of typhoons in eastern and southeastern Asia has increased 2–3 times^2^. This has a serious impact on natural ecosystems, industrial and agricultural production, transportation, and even human livelihoods^3^. In light of the escalating threat of disasters, it is crucial to acquire comprehensive disaster information and investigate the patterns of disaster evolution. This is essential for advancing research in disaster prevention and mitigating the risks associated with disasters^4^. According to “Sendai Framework for Disaster Risk Reduction 2015–2030”, traditional technologies and modern means should be fully utilized to enhance disaster monitoring, risk assessment, and service capabilities through methods such as big data, social media, and mobile internet^5^. Within the realm of disaster big data, a myriad of data types are implicated, with textual data particularly standing out due to its abundance and ubiquity. Accordingly, the extraction of typhoon-related disaster information from textual sources has emerged as a focal point within the domain of emergency management^6,7^.

Event extraction refers to the automatically extracting user interested event information from unstructured text and presenting it in a structured form^8^. The investigation into event extraction related to typhoon disasters is still in its early stage^9,10^. There are two main methods of event extraction: (1) pattern matching method. By formulating the sentence expression pattern of the extracted information, various pattern matching algorithms are used to match the text to be extracted with the extracted template^11^. This method is relatively accurate, but it often relies on specific domain knowledge and has poor portability. (2) machine learning method, especially deep learning. It mainly includes convolutional neural networks^12^, recurrent neural networks^13^, generative adversarial networks^14^, graph convolutional networks^15^, and other machine learning models. The machine learning method regards the event extraction task as a classification problem, focusing on classifier construction and extracting features. It requires less manual intervention and offers good portability. However, the method makes it may struggle to extract general text features and is highly reliant on the corpus, resulting in lower overall precision.

As a typical geographical event, the typhoon disaster has significant spatio-temporal dynamic features^16,17^. Typhoon disaster-oriented event extraction differs from generalized event extraction. It not only identifies the elements inherent in the event (entities, time, attribute values, roles, etc.), but also places heightened emphasis on the entire process of a typhoon disaster, spanning from its start to end. Existing event extraction methods can achieve typhoon disaster detection. For instance, within typhoon disaster texts, occurrences such as <Time1, Place1, infrastructure damage> and <Time2, Place2, airport closure> represent two outcomes of event extraction. However, these fragmented disaster details fail to illustrate the dynamic spatio-temporal evolution of typhoon disasters^18^. There are the following key problems to be solved: (1) at present, there is a lack of information modeling for typhoon disaster process, making it difficult to standardize various information of typhoon disaster process. A systematic information modeling and classification significantly impact the accuracy of information extraction. (2) The process is scale-dependent, and there are differences in the spatial and temporal range and evolution sequence of typhoon disasters at different scales. In emergency management, it is essential to comprehend the processes of typhoon disasters from multiple spatiotemporal scales. Consequently, acquiring information of different granularities to describe these processes is also imperative. (3) Various disaster elements undergo dynamic changes during the process of typhoon disasters. For instance, in the <Time1, Place1, infrastructure damage> , <Time1, Place2, house collapse> , and <Time2, Place2, airport closure> , the subjects of these three event extraction results are infrastructure, houses, and airport respectively. However, the general event extraction method ignores the differences in information representation elements and their hierarchical relationships. Henceforth, this study regards states as the fundamental units for measuring processes, and proposes an information method for typhoon disaster states. The aim is to provide multi-granular information support for analyzing typhoon disaster processes at different spatio-temporal scales in emergency management.

At present, the scarcity of annotated corpora for typhoon disaster states poses challenges in directly applying relevant event extraction methods like pattern matching and machine learning. In addition, the abundance of text expressions featuring the co-occurrence of various typhoon disaster states complicates the extraction process. In response, this paper propose a method of typhoon disaster state information extraction (TDSIE). This method identifies diverse types of information characterizing typhoon disaster states and integrates information from co-referential states. The main contributions include:

1. Leveraging diverse spatio-temporal characteristics, including spatio-temporal information elements, spatio-temporal semantic units, and spatio-temporal clues, this approach extracts typhoon disaster state information from Chinese text. This addresses challenges related to the dispersion of disaster information and the diverse spatio-temporal granularity inherent in conventional disaster information extraction methods.
2. By employing Typhoon Lekima as a case study, the extracted typhoon disaster state information is utilized for multiscale analysis of spatio-temporal patterns and evolution characteristics. This study offers valuable insights into the application scenarios and advantages of the research results.

The main chapters of this paper are as follows: “Related works” introduces the related works, “Basic ideas” explains the basic ideas of typhoon disaster state, “Methodology” proposes the method of TDSIE; “Results and discussion” presents the experimental evaluation and the case study, and “Conclusion” presents the conclusions and future work.

## Related works

The event extraction method needs to rely on the results of natural language processing tasks such as named entity identification, coreference resolution, and relationship extraction, but they are not the focus of the event extraction itself. Presently, the research on event extraction predominantly adheres to the evaluation criteria of conference ACE 2005, comprising four sub-tasks: (1) event trigger identification. This task involves discerning whether the words are event triggers or event types; (2) semantic role labeling. The task is to examine the relationship between various components and the predicate in the event, taking the predicate as the focal point; (3) event attribute classification. The task entails determine event attributes and describe objects; (4) event coreference resolution. The task is to identify whether two event instances belong to the same event^19^.

To realize the event extraction, two methods are mainly used: (1) pattern matching method. The key to this method is the construction of event templates, which is mainly used to indicate the context constraint information that constitutes the target event^20^. The original template construction mainly relies on manual summary, which requires special professional knowledge^21^. Researchers have endeavored to employ machine learning for the automated construction of event templates, demonstrating commendable performance in domain-specific event extraction tasks. However, these templates exhibit limitations in cross-domain applications, leading to suboptimal portability^22^. (2) machine learning method. Based on the statistical model, this method transforms event detection and argument identification into classification problems. The machine learning method can be divided into pipeline method and feature union method. In the pipeline method, since the trigger identification always precedes the argument identification, the event argument cannot be considered when the event trigger identification is performed, which limits the accuracy of the trigger identification^23,24^. Therefore, researchers have proposed the feature union method. The feature union method constructs a joint learning model for tasks such as trigger identification and argument identification, so that trigger word and argument information can promote each other’s extraction effect^25,26^.

In recent times, machine learning methods, particularly deep learning and neural networks, have emerged as primary techniques for event extraction. Fully connected neural networks, convolutional neural networks^27^ and recurrent neural networks^28,29^ have been applied to event extraction. In addition, the weakly supervised method, which can automatically generate tagged corpus and alleviate the problem of data sparsity, is gradually gaining traction in event extraction applications.

Because of the significant spatio-temporal dynamic features of typhoon disasters, it is difficult to achieve the event extraction of typhoon disasters on multiple spatio-temporal nodes. More importantly, the results of events extraction are all static information elements related to typhoon disasters, which are difficult to reflect the dynamic nature of the process of typhoon disaster^30,31^. The state is the detailed representation of a continuous process, and the state of typhoon disaster is the existing form of typhoon disaster under specific spatio-temporal conditions^32^. Thus, the extraction of different states of typhoon disaster becomes the premise to perceive the dynamic characteristics of typhoon disaster. The existing event extraction methods can be used for reference: (1) it is essential to ascertain whether the information within the text constitutes a state element and to determine its type. This is crucial for achieving the identification of the typhoon disaster states. (2) The text may encompass multiple states simultaneously, and a particular state may be referenced multiple times within the text. It is imperative to discern whether the recognized state pertains to the co-referential state of a typhoon disaster under the same spatio-temporal conditions.

Nonetheless, the characteristics of typhoon disaster states present unique challenges that render standard event extraction methods unsuitable for their extraction. (1) The classification of typhoon disaster states exhibits fine granularity and encompasses various types, with certain similarities in the features of state contexts. The application of pattern matching methods can potentially result in trigger template errors, hindering the formulation of accurate matching templates. (2) Supervised machine learning and deep learning heavily depend on extensive labeled corpora. Otherwise, the issue of data sparsity becomes pronounced. Currently, there is a scarcity of tagged Chinese corpora for the states of typhoon disasters, posing a challenge to the effective training of relevant machine learning models. (3) Events, being conceptual units more complex than entities, pose a challenge for existing extraction methods that rely on deep semantic understanding. Presently, deep semantic understanding technology is imperfect, exhibiting low accuracy and lacking universality in open fields. This limitation may impede the information extraction of typhoon disaster states. Hence, it becomes imperative to adeptly integrate the Chinese text description characteristics with the evolution process features of typhoon disasters. This approach is essential for devising a method tailored to the effective information extraction of typhoon disaster states.

## Basic ideas

**Figure 1:**
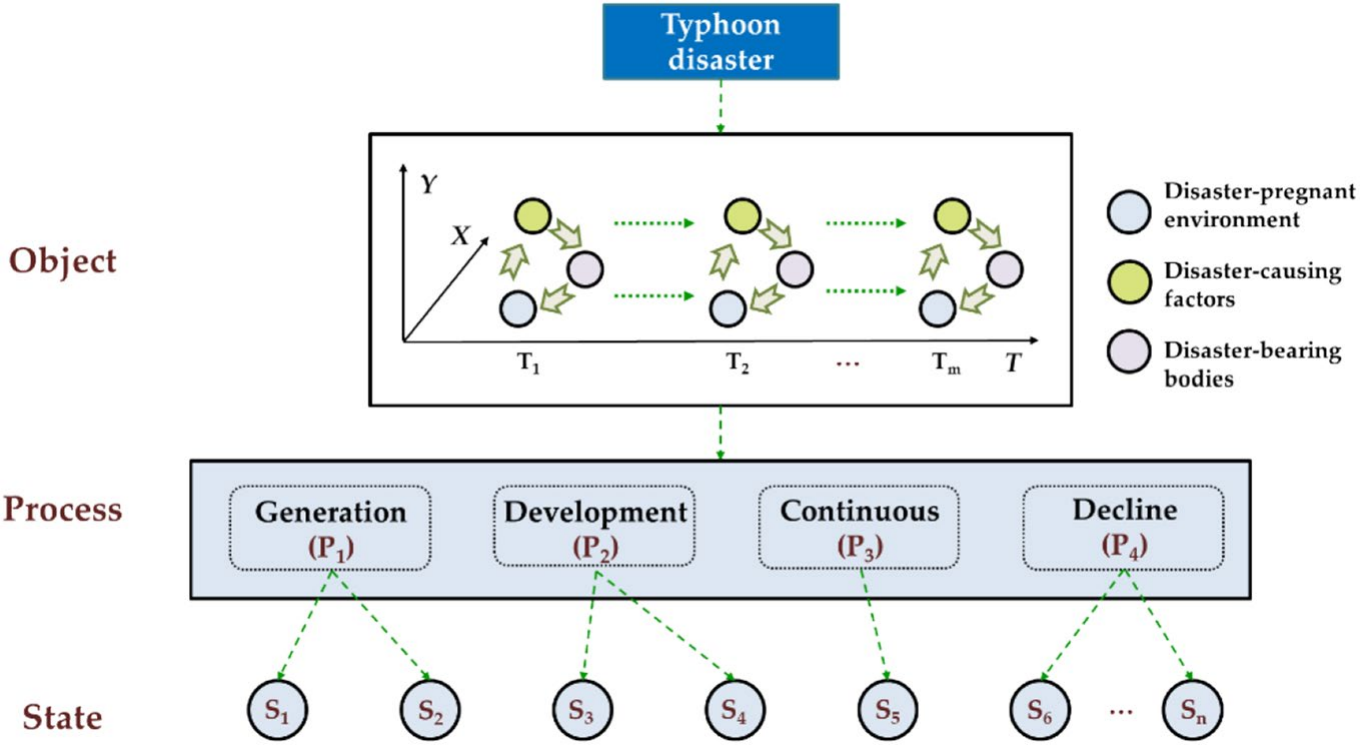
The framework of the typhoon disaster information representation model^33^.

A typhoon disaster constitutes a catastrophic event resulting from a typhoon, giving rise to casualties, economic losses, and environmental damage. Regarding component objects, typhoon disasters encompass the disaster-pregnant environment, disaster-causing factors, and the disaster-bearing body. In terms of dynamic characteristics, the interaction among these components delineates the developmental process of motion. Consequently, information related to typhoon disasters can be categorized into three levels: object level, process level, and state level. The typhoon disaster information representation model is detailed in Fig. 1.

In the representation model of typhoon disaster information, the process directly refers to the lifecycle composed of stages such as initiation, development, persistence, and decay. State sequences constitute the fundamental units of the process. State sequences are formed by connecting states to each other, thus states represent relatively stable forms of existence in the typhoon disaster process. Under specific time and place conditions, combined with the attributes, behaviors and influence, different states are formed.

1. Time and place serve as the basic framework. Time and place describe the spatio-temporal features of states, providing the prerequisite conditions for the existence of other dimensional features. Specifically, time measures the sequence of state occurrences, while place records the spatial or positional context of state occurrences.
2. Attributes and behaviors serve as the core features. Attributes and behaviors describe the intrinsic features of typhoons, representing important manifestations of their developmental stages. Attributes, which are relatively static, depict the morphology of typhoons, while dynamic behaviors illustrate their actions.
3. Influence serves as the peripheral interaction feature. Influence describes the interaction between typhoons and the surrounding environment. The genesis, development, and evolution of typhoons are all influenced by their surrounding environment. Moreover, typhoons can cause significant damage to the surrounding environment after occurrence, all of which fall under the category of influence features.

Furthermore, based on the differences in attribute and behavior features, states can be categorized into different types. States can be regarded as highly generalized representations of various features of typhoon disasters under specific spatio-temporal conditions. For instance, on August 10, 2019, at 1:45 am, Typhoon Lekima was located in Wenzhou city, Zhejiang province, with maximum wind speeds reaching level 16. By August 11, at 8:50 pm, it had moved to Qingdao city, Shandong province, with maximum wind speeds decreasing to level 9. Typhoon Lekima was in two different states at <1:45am on August 10th, Wenzhou city> and <8:50 pm on August 11th, Qingdao city>. If we categorize states based on wind force levels, the types of the two states would be “super typhoon level” and “tropical storm level,” respectively.

In this paper, the structure of the typhoon disaster state is defined as a five-tuple of <time, place, attribute, behavior, influence>. The classification system and coding of typhoon disaster state information are shown in Table 1.

**Table 1:** Classification system and coding of typhoon disaster state information.

First class	Second class	Third class
Time (10)	Direct time (1001)	Second (100101) Minute (100102) Hour (100103) Day (100104) Week (100105)
	Indirect time (1002)	
Place (20)	Direct place (2001)	Landmark (200101) Street (200102) County administrative division (200103) Municipal administrative division (200104) Provincial administrative division (200105)
	Indirect place (2002)	
Attribution (30)	Speed (3001)	Wind strength (300101) Wind speed (300102)
	Strength (3002)	Tropical depression (300201) Tropical storm (300202) Severe tropical storm (300203) Typhoon (300204) Severe typhoon (300205) Super typhoon (300206)
	Temperature (3003)	Air temperature (300301)
	Humidity (3004)	Precipitation (300401) Air humidity (300402)
Behavior (40)	Transfer (4001)	Landing (400101) Move (400102) Leaving land (400103)
	Change (4002)	Formation (400201) Numbering (400202) Upgrading (400203) Continuing (400204) Downgrading (400205) Dissipation (400206) Degeneration (400207) Combination (400208)
Influence (50)	People (5001)	Injury (500101) Death (500102) Missing (500103) …
	Infrastructure (5002)	Destruction (500201) Damage (500202) Collapse (500203) …
	Traffic (5003)	Highway (500301) Steamship (500302) Aircraft (500303) …
	Social activity (5004)	Hospital (500401) School (500402) Factory (500403) …
	Secondary disaster (5005)	Meteorological disaster (500501) Marine disaster (500502) Geological disaster (500503) …

## Methodology

**Figure 2:**
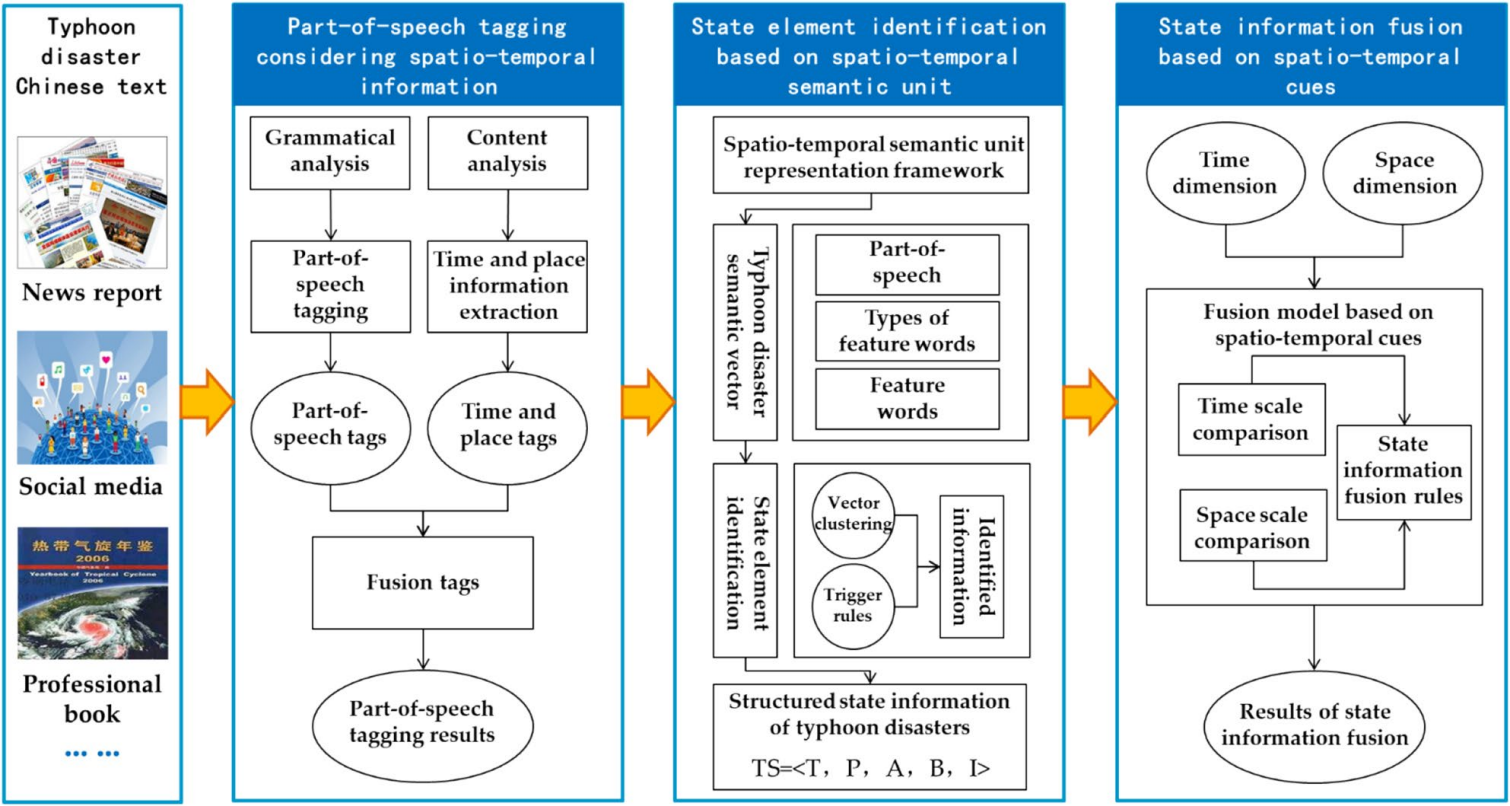
Technical roadmap of the TDSIE.

To extract dynamic typhoon disaster information from extensive text within a big data environment, a method called typhoon disaster state information extraction (TDSIE) is proposed, and its technical framework is depicted in Fig. 2. The TDSIE process unfolds in several steps:

1. Part-of-speech tagging, time, and place extraction.
  These tasks are executed independently, and their results are amalgamated to achieve comprehensive word tagging within the Chinese text.
2. Text segmentation into spatio-temporal semantic units.
  Based on time and place labels, the Chinese text is segmented into distinct spatio-temporal semantic units. The embedding features of word vectors are then extended, incorporating text characteristics specific to the typhoon disaster states. Then, utilizing vector clustering, various elements defining the typhoon disaster states are identified within each spatio-temporal semantic unit.
3. Identification of state coreference relationships.
  Leveraging time and place elements within the states of typhoon disasters as cues, a model is employed to identify coreference relationships across different spatio-temporal semantic units. Subsequently, relevant information pertaining to state coreference is fused.
  This methodology aims to effectively extract and integrate information regarding the typhoon disaster states from massive textual data in a big data environment.

### Part-of-speech tagging considering spatio-temporal information

Part-of-speech tagging entails assigning grammatical categories to words based on their linguistic attributes. However, when it comes to time and place information, conventional part-of-speech tagging often labels them as nouns, pronouns, and similar categories, making it challenging to distinguish them from other types of information. Within the constituent elements defining the state of typhoon disasters, time and place serve as the foundational framework. As such, the spatio-temporal features embedded in Chinese text become crucial for extracting information related to typhoon disaster states. Therefore, it becomes imperative to consider the spatio-temporal semantics of content during the part-of-speech tagging process.

Time and place information extraction can effectively make up for the deficiency of part-of-speech tagging^34,35^. Building upon the initial part-of-speech tagging, the extraction of time and place information is carried out separately. The outcomes of these extractions are then annotated with corresponding time and place tags, subsequently replacing the original tags within the part-of-speech tagging results (Table 2). The meaning and explanation of part of speech^36^, time and place tags are shown in Table 3.

**Table 2:** Example of text tagging.

Tag	Example (in Chinese)	Example text semantics
Part-of-speech tags	今年/n 第9号/m 台风/n “/w 利奇马/n ”/w (/w 热带风暴/n 级/q ) /w 的/u 中心/n 已于/d 11日/n 20时/n 50分/n 前后/f 在/p 青岛市/n 黄岛区/n 沿海/f 登陆/v 。/w	The center of this year ‘s No. 9 typhoon, Typhoon Lekima (tropical storm-level), landed on the coast of Huangdao district, Qingdao city at about 20:50 on November 11
Time and place tags	今年/t 第9号台风“利奇马”(热带风暴级) 的中心已于 11日20时50分/t 前后在 青岛市黄岛区/ns 沿海登陆。	
All tags	今年/t 第9号/m 台风/n “/w 利奇马/n ”/w (/w 热带风暴/n 级/q ) /w 的/u 中心/n 已于/d 11日20时50分/t 前后/f 在/p 青岛市黄岛区/ns 沿海/f 登陆/v 。/w

**Table 3:** Main tags and their meanings.

Tag	Explanation	Tag	Explanation	Tag	Explanation
t	Time	ns	Place	f	Direction
n	Noun	nr	Personal name	nt	Institution name
ng	Noun gender	v	Verb	vg	Verbal gender
vd	adverbial verb	vn	Noun–verb	a	Adjective
d	Adverb	m	Numeral	q	Quantifier
mq	Numeral quantifier	r	Pronoun	u	Auxiliary word
p	Preposition	c	Conjunction	w	Punctuation mark

### State element identification based on spatio-temporal semantic unit

#### Construction of spatio-temporal semantic unit

Words, phrases, clauses, sentences or paragraphs are all linguistic units, and the basic structure of the text is formed through semantic relations between units^37^. If some linguistic units or different linguistic units can be combined to express a complete semantic connotation, it is a semantic unit. For instance, “At 6:00 on August 10, the center of Typhoon Lekima, the ninth typhoon of the year, was located in Huangyan District, Taizhou City, Zhejiang Province” is a semantic unit about a typhoon disaster state.

**Figure 3:**
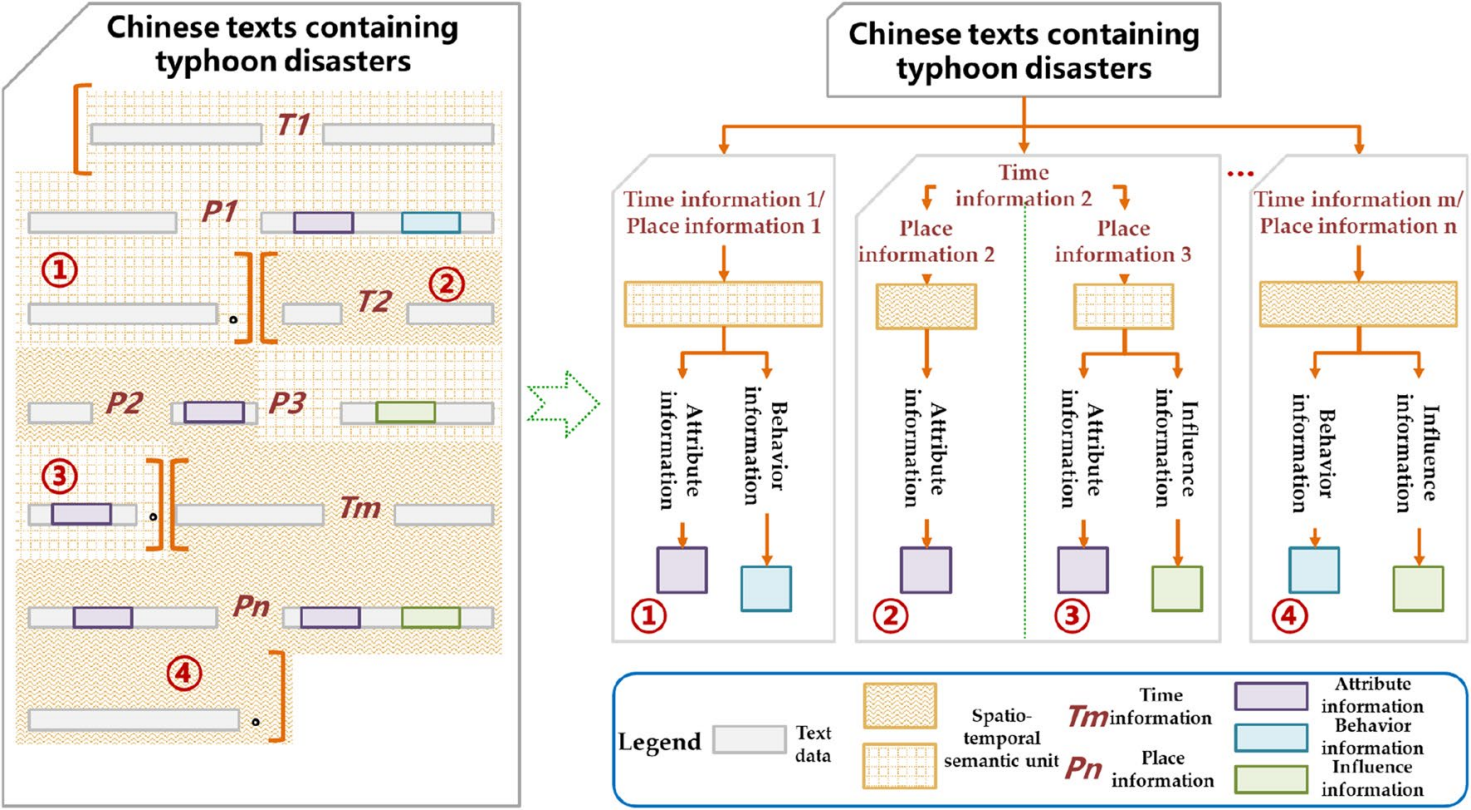
Spatio-temporal semantic unit representation framework in text.

Through text analysis of typhoon events, changes in spatio-temporal information serve as transition markers in describing the state of typhoon disasters. Therefore, the spatio-temporal information can be used to track the state transitions recorded in the text. It is evident that the semantic units of typhoon disaster states contain spatio-temporal information, which is the important basis for classifying the semantic units of typhoon disaster states. Based on the spatiotemporal characteristics of the text, a framework for represnting spatio-temporal semantic units is constructed, and the text is divided into different spatio-temporal semantic units (Fig. 3).

#### State element identification

Each spatio-temporal semantic unit contains various elements about the typhoon disaster states. Since the time and place information has been tagged, the identification of state elements mainly aims at attribute, behavior and influence information. In this paper, an extended semantic vector-based approach for recognizing state elements of typhoon disasters is proposed.

The first step involves extending the feature set of word vector embedding specifically tailored for typhoon disaster states. Currently, word vector construction predominantly focuses on modeling words, grammar, and contextual aspects within text. However, the specific domain features related to typhoons are often overlooked in tasks such as state element identification. To augment discrimination, the extended feature set for word vector embedding in this study incorporates feature words, feature word types, and part-of-speech information. This comprehensive approach aims to enhance the representation of typhoon-related content and improve the accuracy of state element identification.

- Feature words and their types. By sorting out the semi-structured typhoon event knowledge provided in the form of information boxes in the online encyclopedia knowledge base, several different types of typhoon disaster state feature words are summarized and refined to form a feature lexicon (Table 4). The word *w*_*i*_ in the text is matched with the feature lexicon, and the lexical tag is encoded *r*_*i*_ according to the different types of matching. If all the words do not match, *r*_*i*_ = 0.
- Part of speech. As a basic grammatical attribute, part of speech can judge the component words act as in the text. According to the tagging results, the part of speech of the tagged words is si.
  The words are combined with various extended features to form the word-feature pairs < *w*_*i*_–*r*_*i*_–*s*_*i*_ > . Word-feature pairs are used to train word vectors, and each word is mapped to a k-dimensional real number vector to get the typhoon disaster semantic vector *v*_*i*_.
  The second step is trigger word generation based on extended vector clustering. The typhoon disaster state needs to be characterized by a series of state elements, and the high-frequency words that appear repeatedly in the text are more likely to be state elements. In particular, because the semantics of numerals and quantifiers are clear to distinguish, the trigger words of state elements of typhoon disasters are mined from nouns and verbs. (a) The word frequency statistics of nouns and verbs appearing in the text are carried out, and the high-frequency words are selected to form a candidate set. (b) The hierarchical clustering method is used to cluster the words in the candidate set. Based on the typhoon disaster semantic vector *v*_*i*_, the semantic similarity can be determined by the cosine distance between the vectors:
  1 $${\text{cos}}\left({w}_{i},{w}_{j}\right)=\frac{\sum_{k=1}^{N}({w}_{i}^{k}\times {w}_{j}^{k})}{\sqrt{\sum_{k=1}^{N}{({w}_{i}^{k})}^{2}}\times \sqrt{\sum_{k=1}^{N}{({w}_{j}^{k})}^{2}}}$$
  In the formula, ${v}_{i}^{k}$ denotes the *k*th dimension of the word vector of the word *w*_*i*_, and *N* denotes the dimension of the vector.
- The noise of the clustering results is filtered, includes: non-feature class cluster, and the whole class cluster has no feature words in Table 4; the non-triggering candidate words in the characteristic cluster. The similarity between the center word of the cluster and each candidate word in the cluster is calculated, and the candidate words with great differences in similarity are filtered out. Then, the filtered clusters are calculated for similarity with different types of feature words in Table 4, and the clusters are divided into the feature types with the highest similarity to form the attribute, behavior and influence trigger lexicon of typhoon disaster states.

**Table 4:** Feature words of state elements of typhoon disasters.

Element	Feature words		Encode
Attribute	wind speed, wind speed, maximum wind speed, average wind speed, central wind, central pressure, radius of wind circle, typhoon grade, grade, cloud diameter, cloud radius, eye, eye area, super typhoon, strong typhoon, typhoon, strong tropical storm, tropical storm, tropical depression, etc		1
Behavior	generation, coding, naming, landing, upgrading, persistence, degradation, dissipation, merger, transformation, suspension, etc		2
Influence	Casualty	death, disappearance, injury, disaster, loss of life, casualty, serious injury, minor injury, number of casualties, number of deaths, number of casualties, number of casualties, situation of casualties, situation of disappearance, affected population, etc	3
	Economic loss	loss, disaster loss, property loss, economic loss, agricultural loss, infrastructure loss, crop lodging, field collapse, affected area, etc	
	Facilities damage	collapse, damage to houses, outage, suspension, highway closure, return to the port to avoid wind, power failure, water stop, gas stop, etc	
	Secondary disaster	debris flow, rainstorm, huge wave, storm surge, tsunami, flood, waterlogging, flash flood, etc	
	Emergency rescue	transfer, resettlement, rescue, shelter, asylum, refuge, work stoppage, class suspension, production stoppage, reinforcement inspection, investigation, procurement, emergency response, etc	

The third step involves the identification of state elements through the fusion of trigger words and rules. The expression forms of state element information include two types: key-value type and representation type. The key-value type presents the form of “element name → element value”, while the representation type directly expresses the element value without prompting the element name. For key-value type, not only the element information needs to be identified, but also the relationship between “element name → element value” needs to be further extracted. The state element information has the following distribution law: (a) co-occurrence law: element names and element values often appear in the same sentence; (b) positional law: element names and element values are positionally adjacent, and their positional distributions are usually not more than three words; (c) part of speech law: element values are often composed of “numerals + quantifiers “. Due to the territoriality of typhoon texts, the applicable quantifiers are also relatively fixed, it mainly includes kilometers, meters, millimeters, meters/seconds, kilometers/hour, level, hectopascal, etc. Through pattern matching of text and trigger rules, the state elements of typhoon disasters are identified (Table 5). Furthermore, the type of state elements (attribute, behavior, and influence) is determined based on the category of trigger words.

**Table 5:** Trigger rules for state elements of typhoon disasters.

Element	Rule	Element	Rule
Attribute	attribute trigger word + /m|/mq	Influence	/n|/nr|/nt + influence trigger word
	attribute trigger word + /q		influence trigger word + /n
	attribute trigger word + /m|/mq + /q		/n|/nr|/nt + /v + influence trigger word
	/m|/mq + attribute trigger word		influence trigger word + /n + /v
	/m|/mq + /q + attribute trigger word		/v + /v + influence trigger word
	attribute trigger word + /v + /m|/mq + /q		influence trigger word + /v + /v
	/m|/mq + /q + /v + /m|/mq + /q + attribute trigger word		influence trigger word + /m|/mq
Behavior	behavior trigger word		influence trigger word + /q
	/a + behavior trigger word		influence trigger word + /m|/mq + /q
	/d + behavior trigger word		influence trigger word + /v + /m|/mq + /q
Influence	/m|/mq + influence trigger word		/m|/mq + /q + /v + /m|/mq + /q + influence trigger word
	/m|/mq + /q + influence trigger word		/n + /cc + /n + influence trigger word

#### Structured representation of state elements

According to the tuple structure of typhoon disaster states, the typhoon disaster state is reconstructed through the process of slot filling. Formally, the typhoon disaster state can be represented as *TS*:

2 $$TS=<T, P, A, B,I>$$

In the formula, *T* is the time element, *P* is the place element, *A* is the attribute element, *B* is the behavior element and *I* is the influence element.

**Figure 4:**
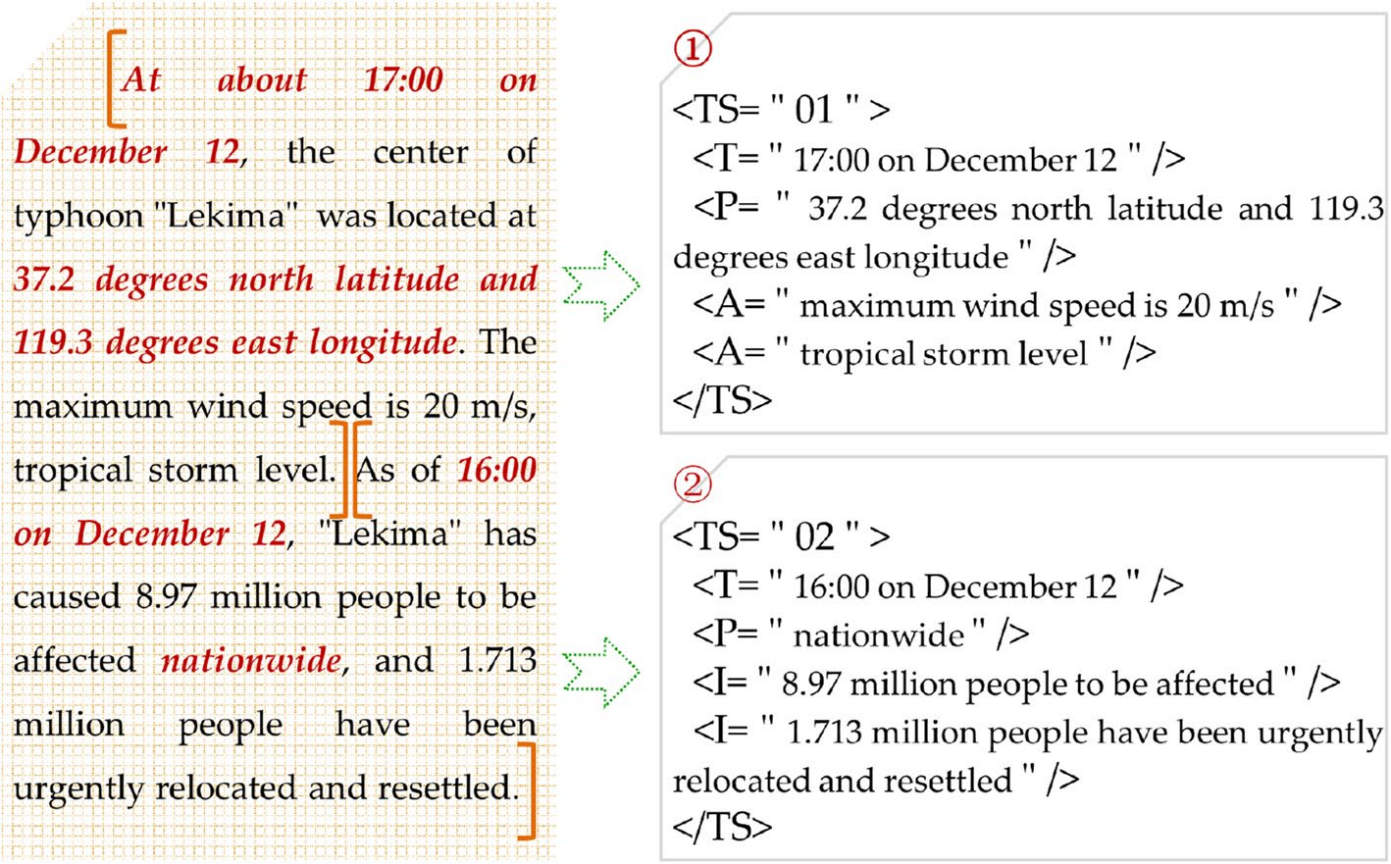
Structured results of typhoon disaster state information.

Because the text has been divided into many spatio-temporal semantic units, the information of state elements of typhoon disasters is distributed in each unit. Therefore, the information of state elements can be structured according to the units they belong to. In each spatio-temporal semantic unit, the time and place information of part-of-speech tagging and the identified attributes, behaviors and influence information are filled according to the slot of formula (2) (Fig. 4). The description of typhoon disaster state in a spatio-temporal semantic unit may be limited to a certain aspect, and the attribute, behavior and influence elements may be missing in the structural representation.

### State information fusion based on spatio-temporal cues

The same state of a typhoon disaster may be repetitively mentioned at various locations within the text. The spatio-temporal scales of different typhoon disaster states are also inconsistent. Moreover, certain types of state elements may be absent, resulting in incomplete and semantically ambiguous information obtained through state extraction^38^. Therefore, after identifying the elements of typhoon disaster states, further integration is required for co-referential information pertaining to typhoon disaster states. On one hand, aligning the spatio-temporal scales and element content of different states is possible. On the other hand, efforts should be made to avoid redundant information in co-referential states.

Typhoon disaster states exist in a certain range of time and space, and time and place become an important basis for distinguishing different typhoon disaster states. It is worth noting that the information of time and place in the text is often inconsistent and incomplete, and a lot of information cannot be fused directly by hard matching. This paper takes time and space as a clue to explore the important features of co-referentiality among multiple spatio-temporal semantic units. In the fusion process, it is assumed that the typhoon disaster states *TS*_1_ and *TS*_2_, <*T*_1_, *P*_1_, *A*_1_, *B*_1_, *I*_1_> and <*T*_2_, *P*_2_, *A*_2_, *B*_2_, *I*_2_> are the elements of the typhoon disaster states, and the relevant rules in Table 6 are followed.

**Table 6:** Fusion rules of state information based on spatio-temporal cues.

Type	T_1_ = T_2_?		P_1_ = P_2_?		TS_1_ = TS_2_?	Fusion results	Instantiation
Fusion rules of “same time element + same place element”(R1)	*T*_1_ = *T*_2_		*P*_1_ = *P*_2_		The element names are the same, and the element values are the same or compatible	The element values with high precision in *TS*_1_ and *TS*_2_ are taken as the fusion results	*TS*_1_ = <*T*: August 11th, *P*: Qingdao City, Shandong Province, *B*: landfall>, *TS*_2_ = <*T*: August 11th, *P*: Qingdao City, Shandong Province, *B*: landfall again>. The fusion result of *TS*_1_ and *TS*_2_ is *TS*_*u*_ = <*T*: August 11th, *P*: Qingdao City, Shandong Province, *B*: landfall again>
					The element names are the same, but the element values are different or incompatible	*TS*_1_ and *TS*_2_ are not coreferential and do not fuse state information	*TS*_1_ = <*T*: August 11th, *P*: Qingdao City, Shandong Province, *A*: The central wind speed is 23 m/s>, *TS*_2_ = <*T*: August 11th, *P*: Qingdao City, Shandong Province, *A*: The central wind speed is 25 m/s>. Then *TS*_1_ and *TS*_2_ are not fused
					The element names are different	The different elements in *TS*_1_ and *TS*_2_ are all taken as the fusion results	*TS*_1_ = <*T*: August 11th, *P*: Qingdao City, Shandong Province, *B*: landfall>, *TS*_2_ = <*T*: August 11th, *P:* Qingdao City, Shandong Province, *A*: The central wind speed is 23 m/s>. The fusion result of *TS*_1_ and *TS*_2_ is *TS*_*u*_ = <*T*: August 11th, *P:* Qingdao City, *A*: The central wind speed is 23 m/s, *B:* landfall>
Fusion rules of “same time element + different scale of place element”(R2)	*T*_1_ = *T*_2_		*P*_1_ and *P*_2_ scales are different	*P*_1_ and *P*_2_ are subordinate	Element names are the same in *TS*_1_ and *TS*_2_, and element values are the same or compatible	The element values in the *TS* of the smaller scale *L* are taken as the fusion results	*TS*_1_ = <*T*: August 11th, *P*: Qingdao City, Shandong Province, *B*: landfall>, *TS*_2_ = <*T*: August 11th, *P*: Huangdao District, Qingdao City, Shandong Province, *B*: landfall again>. The fusion result of *TS*_1_ and *TS*_2_ is *TS*_*u*_ = <*T*: August 11th, *P*: Huangdao District, Qingdao City, Shandong Province, *B*: landfall again>
					Element names are the same in *TS*_1_ and *TS*_2_, but element values are different or incompatible	*TS*_1_ and *TS*_2_ are not coreferential and do not fuse state information	*TS*_1_ = <*T*: August 11th, *P*: Qingdao City, Shandong Province, *A*: The central wind speed is 23 m/s>, *TS*_2_ = <*T*: August 11th, *P*: Huangdao District, Qingdao City, Shandong Province, *A:* The central wind speed is 25 m/s>. Then *TS*_1_ and *TS*_2_ are not fused
					Element names are different in *TS*_1_ and *TS*_2_	The element values in the *TS* of the larger scale *P* are taken as the fusion results	*TS*_1_ = <*T*: August 11th, *P*: Qingdao City, Shandong Province, *B*: landfall>, *TS*_2_ = <*T*: August 11th, *P*: Huangdao District, Qingdao City, Shandong Province, *A*: The central wind speed is 23 m/s>. The fusion result of *TS*_1_ and *TS*_2_ is *TS*_*u*_ = < *T*: August 11th, *P*: Qingdao City, Shandong Province, *B*: landfall>
				*P*_1_ and *P*_2_ have no subordination	–	*TS*_1_ and *TS*_2_ are not coreferential and do not fuse state information	*TS*_1_ = <*T*: August 11th, *P*: Qingdao City, Shandong Province, *A*: The central wind speed is 23 m/s> , *TS*_2_ = <*T*: August 11th, *P*: Jilin Province, *I*: Heavy-hard rain>. Then *TS*_1_ and *TS*_2_ are not fused
Fusion rules of “different scale of time element + same place element”(R3)	*T*_1_ and *T*_2_ scales are different	*T*_1_ and *T*_2_ are subordinate	*P*_1_ = *P*_2_		Element names are the same in *TS*_1_ and *TS*_2_, and element values are the same or compatible	The element values in the *TS* of the smaller scale *T* are taken as the fusion results	*TS*_1_ = <*T*: August 11th, *P*: Qingdao City, Shandong Province, *B*: landfall>, *TS*_2_ = <*T*: 20:00 on August 11th, *P:* Qingdao City, Shandong Province, *B*: landfall again>. The fusion result of *TS*_1_ and *TS*_2_ is *TS*_*u*_ = < *T*: 20:00 on August 11th, *P*: Qingdao City, Shandong Province, *B:* landfall again>
					Element names are the same in *TS*_1_ and *TS*_2_, but element values are different or incompatible	*TS*_1_ and *TS*_2_ are not coreferential and do not fuse state information	*TS*_1_ = <*T*: August 11th, *P*: Qingdao City, Shandong Province, *A*: The central wind speed is 23 m/s> , *TS*_2_ = <*T*: 20:00 on August 11th, *P*: Qingdao City, Shandong Province, *A*: The central wind speed is 25 m/s> . Then *TS*_1_ and *TS*_2_ are not fused
					Element names are different in *TS*_1_ and *TS*_2_	The element values in the *TS* of the larger scale *T* are taken as the fusion results	*TS*_1_ = <*T*: August 11th, *P*: Qingdao City, Shandong Province, *B*: landfall> , *TS*_2_ = <*T*: 20:00 on August 11th, *P:* Qingdao City, Shandong Province, *A:* The central wind speed is 23 m/s> . The fusion result of *TS*_1_ and *TS*_2_ is *TS*_*u*_ = <*T*: August 11th, *P:* Qingdao City, Shandong Province, *B*: landfall>
		*T*_1_ and *T*_2_ have no subordination			-	*TS*_1_ and *TS*_2_ are not coreferential and do not fuse state information	*TS*_1_ = <*T*: August 11th, *P*: Qingdao City, Shandong Province, *A*: The central wind speed is 23 m/s> , *TS*_2_ = <*T*: August 12th, *P*: Qingdao City, Shandong Province, *I*: Heavy-hard rain> . Then *TS*_1_ and *TS*_2_ are not fused
Fusion rules of “different scale of time element + different scale of place element”(R4)	*T*_1_ and *T*_2_ scales are different	*T*_1_ and *T*_2_ are subordinate	*P*_1_ and *P*_2_ scales are different	*P*_1_ and *P*_2_ are subordinate	Element names are the same in *TS*_1_ and *TS*_2_, and element values are the same or compatible	The element values with higher precision in *TS*_1_ and *TS*_2_ are taken as the fusion results	*TS*_1_ = <*T*: August 11th, *P*: Qingdao City, Shandong Province, *B*: landfall> , *TS*_2_ = <*T*: 20:00 on August 11th, *P:* Huangdao District, Qingdao City, Shandong Province, *B*: landfall again> . The fusion result of *TS*_1_ and *TS*_2_ is *TS*_*u*_ = <*T*: 20:00 on August 11th, *P*: Huangdao District, Qingdao City, Shandong Province, *B*: landfall again>
					Element names are the same in *TS*_1_ and *TS*_2_, but element values are different or incompatible	*TS*_1_ and *TS*_2_ are not coreferential and do not fuse state information	*TS*_1_ = <*T*: August 11th, *P*: Huangdao District, Qingdao City, Shandong Province, *A*: The central wind speed is 23 m/s> , *TS*_2_ = <*T*: 20:00 on August 11th, *P*: Qingdao City, Shandong Province, *A*: The central wind speed is 25 m/s> . Then *TS*_1_ and *TS*_2_ are not fused
					Element names are different in *TS*_1_ and *TS*_2_	The element values with lower precision in *TS*_1_ and *TS*_2_ are taken as the fusion results	*TS*_1_ = <*T*: August 11th, *P*: Huangdao District, Qingdao City, Shandong Province, *B*: landfall> , *TS*_2_ = <*T*: 20:00 on August 11th, *P*: Qingdao City, Shandong Province, *A*: The central wind speed is 23 m/s> . The fusion result of *TS*_1_ and *TS*_2_ is *TS*_*u*_ = <*T*: August 11th, *P*: Qingdao City, Shandong Province, *A*: The central wind speed is 23 m/s, *B*: landfall>
		*T*_1_ and *T*_2_ are subordinate		*P*_1_ and *P*_2_ have no subordination	-	*TS*_1_ and *TS*_2_ are not coreferential and do not fuse state information	*TS*_1_ = <*T*: August 11th, *P*: Qingdao City, Shandong Province, *A*: The central wind speed is 23 m/s> , *TS*_2_ = <*T*: 20:00 on August 11th, *P*: Jilin Province, *I*: Heavy-hard rain > . Then *TS*_1_ and *TS*_2_ are not fused
		*T*_1_ and *T*_2_ have no subordination		*P*_1_ and *P*_2_ are subordinate			*TS*_1_ = <*T*: August 11th, *P*: Huangdao District, Qingdao City, Shandong Province, *A*: The central wind speed is 23 m/s> , *TS*_2_ = <*T*: August 12th, *P*: Qingdao City, Shandong Province, *I*: Heavy-hard rain> . Then *TS*_1_ and *TS*_2_ are not fused
		*T*_1_ and *T*_2_ have no subordination		*P*_1_ and *P*_2_ have no subordination			*TS*_1_ = <*T*: August 11th, *P*: Qingdao City, Shandong Province, *A*: The central wind speed is 23 m/s> , *TS*_2_ = <*T*: August 12th, *P*: Jilin Province, *I*: Heavy-hard rain> . Then *TS*_1_ and *TS*_2_ are not fused

In Table 6, the same element name means the same type of state element in *TS*_1_ and *TS*_2_. For instance, if both *TS*_1_ and *TS*_2_ have attribute elements about wind scale, then *TS*_1_ and *TS*_2_ have the same element names. Element value equality is applicable to text-type element values; if two text-type element values exhibit a reference relationship, they are deemed equal. For instance: *B*_1_ is wind enhancement, and *B*_2_ is grade enhancement. The semantic meaning refers to the typhoon level rising, then element values of *B*_1_ and *B*_2_ are equal. Element value compatibility pertains to numeric-type element values; if two numeric-type element values share an inclusion relationship within the numerical range, they are considered compatible. For instance, *A*_1_ is the wind speed of 30.2 m/s, and *A*_2_ is the wind speed of 30.23 m/s. From the perspective of data accuracy, *A*_2_ ⊆ *A*_1_, then element values of *A*_1_ and *A*_2_ are compatible.

## Results and discussion

### Experimental environment

Compared to traditional official disaster statistics, online news not only provides access to more sources of typhoon event information beyond official media but also exhibits the following characteristics:

1. Timeliness: various news media utilize the internet as a medium for instant information dissemination, offering a convenient channel for the publication and dissemination of typhoon event information.
2. Massive volume: numerous online news sources swiftly aggregate and spread during or after typhoon events, increasingly manifesting characteristics of big data in terms of information quantity, dissemination speed, content diversity, and application value.
3. Diversity: news coverage of typhoon events encompasses various aspects such as typhoon development, weather conditions, disaster situations, and rescue operations. Online news has become an essential media resource that cannot be ignored in the field of disaster management.

Major domestic websites, such as People’s Daily Online, CCTV News, The Paper, and the China Meteorological Administration portal website, have dedicated columns for special series reporting, covering official media, information platforms, and government portal websites. In this study, 1012 news reports on Typhoon Lekima from August 7th to August 13th, 2019, are selected as experimental data.

In the experiment, the accuracy of the state element identification and the effect of state information fusion are analyzed. The ANSJ tool (https://github.com/NLPCHINA/ansj_seg) is used for word segmentation, part-of-speech tagging, and time and place information extraction. Based on the Word2Vec vector (https://github.com/NLPchina/Word2VEC _java), the experimental results of typhoon disaster semantic vector are compared to verify the contribution of extended embedding features in the word vector to state element identification in this method. Compared with the results of information fusion based on text similarity, the effect of spatio-temporal features as clues in state information fusion is verified. In the experiment, the dimension of the word vector is 200, and the CBOW model is used to train the word vector with a window size of 5. The distance between clusters is not less than 0.5 in hierarchical clustering. The evaluation of text similarity adopts the Levenshtein Distance^39^, with a similarity threshold of [0.7, 1).

The precision rate (P), recall rate (R) and F1 value are selected as the evaluation indexes of the state element identification effect, and the precision rate (P) is used as the evaluation index of the state information fusion effect.

3 $${\text{P}}\left({\text{i}}\right)=\frac{{N}_{TP,i}}{{N}_{FP,i}+{N}_{TP,i}}\times 100\%$$

4 $${\text{R}}\left({\text{i}}\right)=\frac{{N}_{TP,i}}{{N}_{TP,i}+{N}_{FN,i}}\times 100\%$$

5 $${\text{F}}1\left({\text{i}}\right)=\frac{2\times P(i)\times R(i)}{P\left(i\right)+R(i)}\times 100\mathrm{\%}$$

In the formula, *N*_*TP,i*_ represents the number of correctly judged samples in class *i*; *N*_*FP,i*_ represents the number of samples mistakenly classified as class *i*; *N*_*FN,i*_ represents the number of *i*-th class samples that are mistakenly classified as other classes.

### Experimental results

#### Comparison of state identification effect

**Figure 5:**
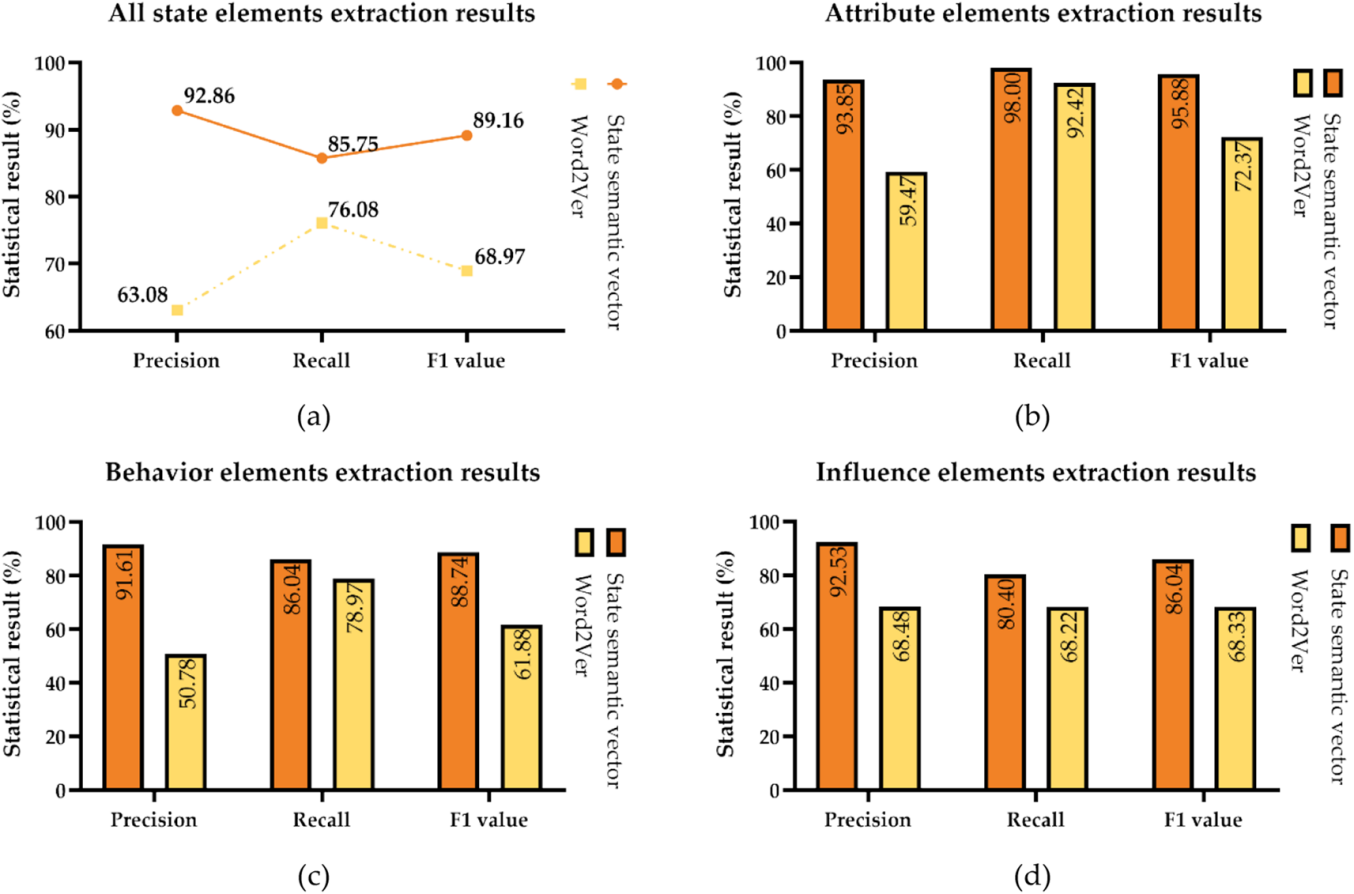
Comparison of state extraction effects. (**a**) Overall result of state extraction; (**b**) extraction result of attribute elements of states; (**c**) extraction result of behavior elements of states; (**d**) extraction result of influence elements of states.

To assess the impacts of different word vector models, the experimental texts is randomly divided into five groups. Four of these groups serve as training data for word vector models, while the remaining one is designated as the test data. The final identification result is derived from the average value obtained through cross-validation. Figure 5 illustrates the precision (P), recall (R), and F1 values for various types of state elements related to typhoon disasters. In this experiment, the Word2Vec is chosen as the baseline method, and it is compared with the state semantic vector proposed in this study.

The experimental results show that the state elements can be identified accurately based on typhoon disaster semantic vector, and the spatio-temporal semantic unit framework establishes the relationship between different state elements and forms structured typhoon disaster state information. The overall extraction precision of attribute elements is higher than that of other elements, reaching P = 93.85%, R = 98.00%. This is because the attribute types of the typhoon disaster state are limited and easier to express explicitly in text. In contrast, the identification of the influence elements is not good, with a recall rate of just 80%. Typhoons will have a destructive impact on the natural environment, social activities and other aspects. The existing types of influence feature words based on the online encyclopedia knowledge base are not comprehensive, which makes it impossible to correctly judge the missing types of related words in word vector training and vector clustering. The identification effect can be improved by increasing the type of feature words and expanding the scale of training text.

Comparing the identification effects under different input vectors, the precision of the identification results based on typhoon disaster semantic vector is significantly better than the Word2Ver, and the recall rate is also improved to a certain extent, resulting in a corresponding increase in the F1 value. Word2Ver reflects the importance of words in a sentence and their implicit relationships with other words. However, these features are still relatively sparse, ignoring the semantic features of typhoon disaster states contained in relevant words. The typhoon disaster semantic vector can well integrate the typhoon disaster state features and context characteristics of the words, and the precision of various state elements is generally balanced. In general, TDSIE uses unsupervised methods to reduce the processing cost of typhoon disaster corpus, and achieves a more accurate identification effect by mining the spatio-temporal features and language knowledge in the text.

#### Comparison of state fusion effect

**Figure 6:**
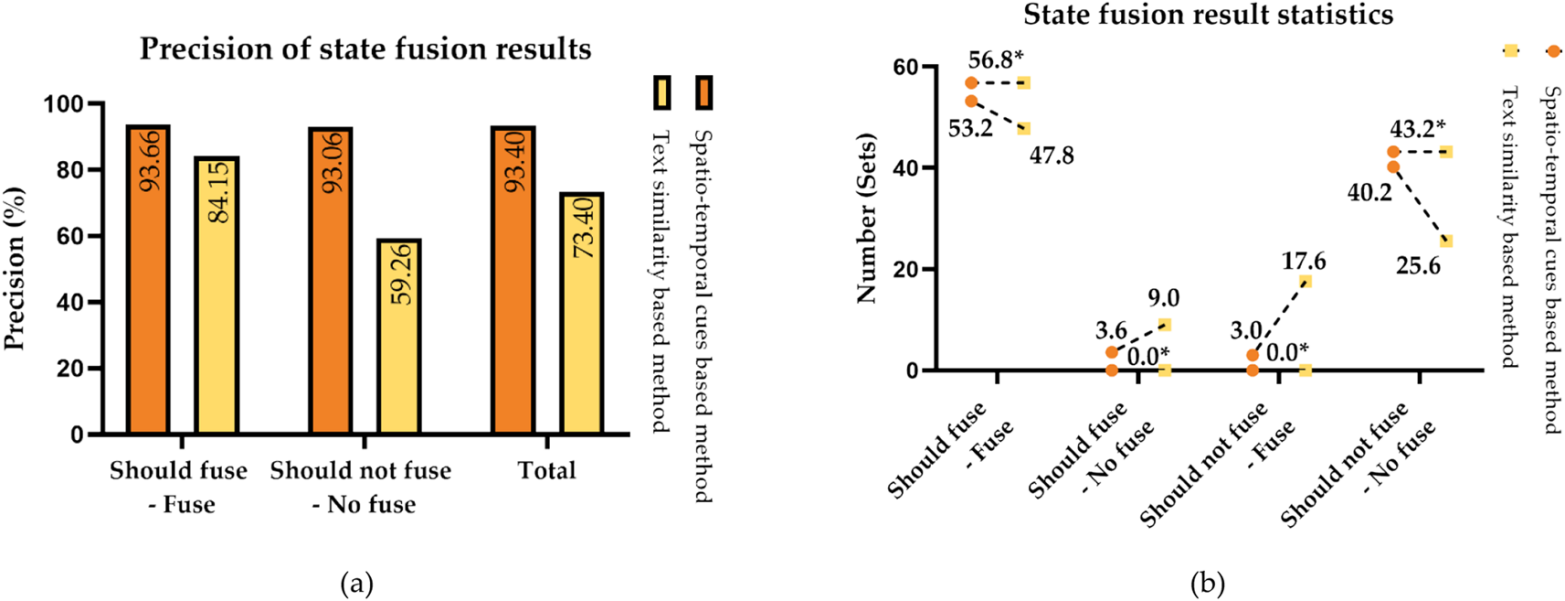
Comparison of state information fusion effect. (**a**) Precision of state fusion results; (**b**) Statistic of state fusion results. In the figure, the meaning of “should fuse-fuse” is that the two states are co-referential and should be fused, which is indeed fused in the experimental results, the others and so on. Besides, (**b**) the data with asterisk (e.g. 56.8*) are the accurate values of manual verification, and the data without asterisk are the statistical results of real experiments.

200 extracted typhoon disaster states are randomly selected, forming 100 sets of state pairs for information fusion. Randomly selecting a total of five iterations, the average value after verification is considered as the final fusion result. Figure 6 displays the precision (P) and numerical statistics of different fusion methods in this experiment. In this experiment, the text similarity-based method is chosen as the baseline method, and it is compared with the spatio-temporal cues-based method proposed in this study.

The results show that there are 93.4 sets of state pairs correctly merged by the fusion method based on spatio-temporal cues, with a precision rate of 93.40%, and all indicators are higher than those based on the text similarity method. It is effective to use spatio-temporal features as the key strategy for state information fusion. The change of time and space is the fundamental sign of state transition. It is not accurate to judge whether the state changes by the change of attribute, behavior or influence.

Analyze the typhoon disaster states that are fused incorrectly in the experiment, mainly due to two aspects: (a) coreference resolution error. The relevant time and place information is not accurately disambiguated. For instance, place information “Jilin” does not accurately resolve whether it is “Jilin Province” or “Jilin City, Jilin Province”; (b) The state element identification error, or the state elements are incomplete. This will lead to the lack of key information on the typhoon disaster states, and thus cannot correctly analyze the fusion results.

#### Experimental result analysis

The TDSIE method exhibits precise and well-balanced identification of various typhoon disaster state elements, resulting in an average 29% increase in the F1 value. Moreover, it accurately combines co-referential states, demonstrating the effectiveness of TDSIE. TDSIE does not rely on extensive tagging corpora for model training, nor does it necessitate deep semantic analysis of text details. Therefore, it achieves accurate typhoon disaster state information extraction. The scope of information extraction is typhoon disaster information in Chinese text that conforms to the state tuple structure, without limitations to a specific type of state, making TDSIE a universal approach.

### Case study

In the present big data environment, extracting disaster information from text serves as an effective supplement to traditional disaster monitoring methods. TDSIE can proficiently extract information regarding the state of typhoon disasters across various spatio-temporal scales, effectively addressing challenges related to the dispersion of disaster information and spatio-temporal granularity diversity. Conducting quantitative classification based on the results of typhoon disaster state information extraction enables the detection of dynamic characteristics within the typhoon disaster process and provides a comprehensive global reference. This paper utilizes the results of typhoon disaster state information extraction from August 10 to 12, 2019, as a case study.

#### Typhoon disaster states at various spatio-temporal scales

**Figure 7:**
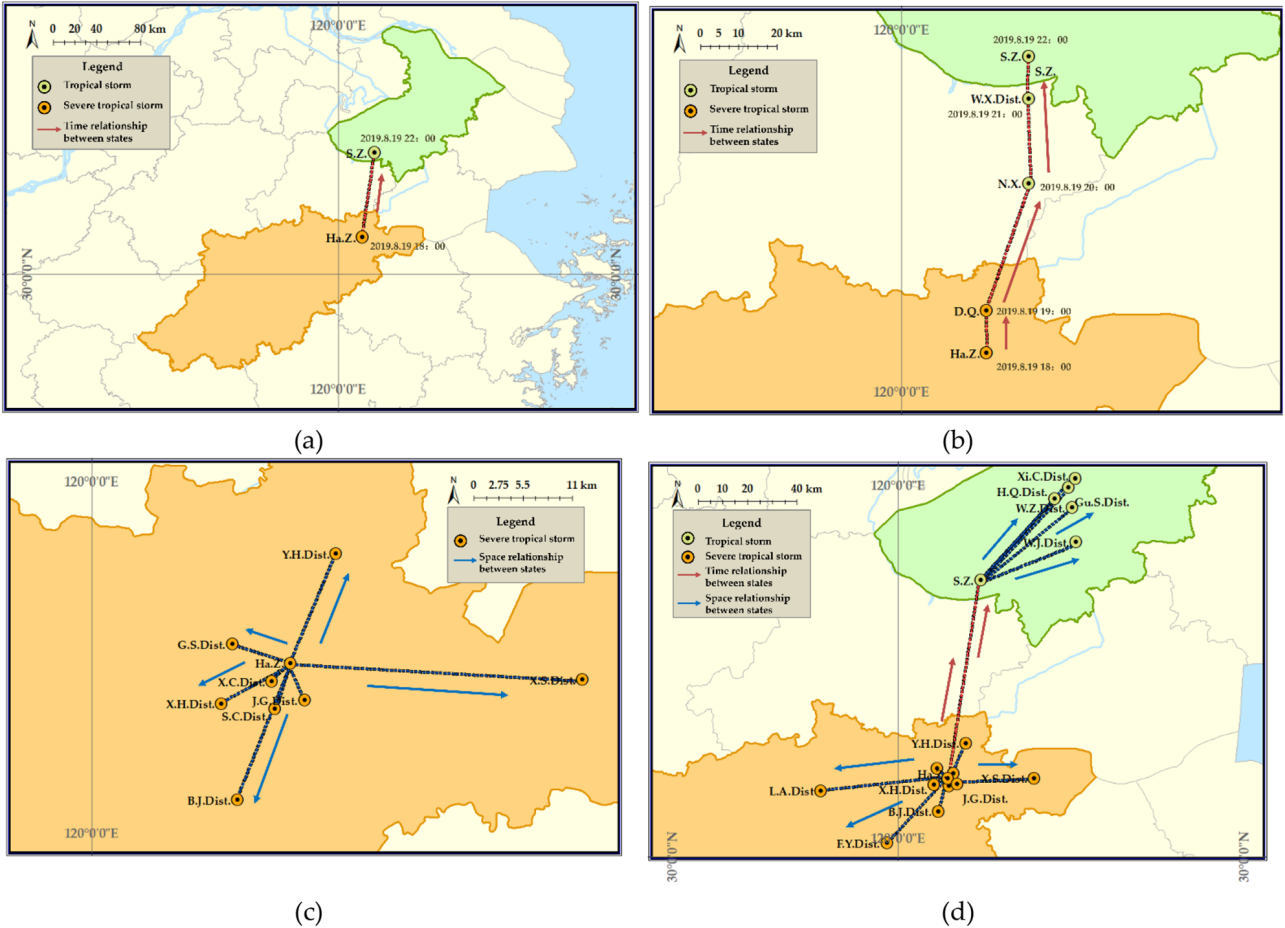
The states and their relationships of typhoon disasters at different spatio-temporal scales. (**a**) Typhoon disaster states with a 4 h temporal scale and a municipal level spatial scale; (**b**) typhoon disaster states with a 1 h temporal scale; (**c**) typhoon disaster states with a county level spatial scale; (**d**) typhoon disaster states with a smaller spatio-temporal scale. The map is self-drawn by the authors, using ArcMap 10.2 sofware (URL: https://www.arcgis.com/index.html).

The abstraction and description of typhoon events across various granularities provide extensive support for disaster early warning, monitoring, command, evaluation, and other decision-making processes. For instance, when analyzing the development trend of a typhoon at the municipal scale, extracting the typhoon disaster state information for different cities at a specific time becomes crucial. To meet the demands of more detailed analysis, reducing the temporal scale enables the examination of typhoon disaster state changes in shorter time intervals. Similarly, reducing the spatial scale facilitates the assessment of disparities in typhoon disaster states among various districts and counties within the city (Fig. 7).

#### Spatial patterns of typhoon disaster states

**Figure 8:**
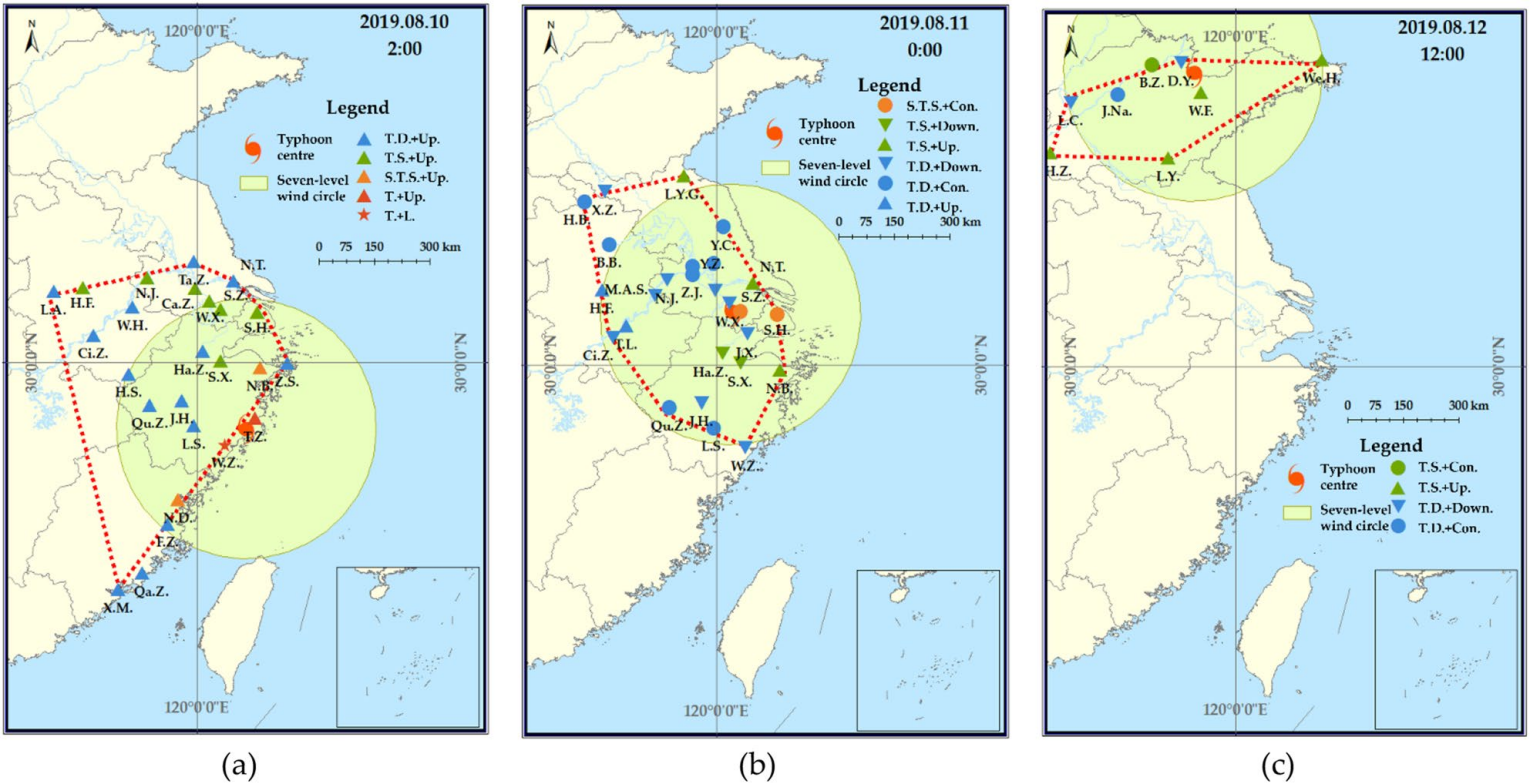
The state changes of different cities in the process of Typhoon Lekima. In the legend, *TD* tropical depression, *TS* tropical storm, *STS* severe tropical storm, *T* typhoon, *L* landing, *Up* upgrading, *Con* continuing, *Down* downgrading. The map is self-drawn by the authors, using ArcMap 10.2 sofware (URL: https://www.arcgis.com/index.html).

The typhoon disaster state information for each city was assessed at a temporal scale of 1 h and a spatial scale at the municipal level (Fig. 8). Throughout the period spanning from August 10 to 12, 2019, corresponding to the advancement of Typhoon Lekima’s life cycle, the impact range of the typhoon exhibited a general pattern of expansion followed by contraction. In comparison with the typhoon track and wind circle issued by the China Meteorological Administration, the distribution range of the detected typhoon state appears more irregular. This suggests non-uniform wind force within the typhoon’s wind circle in different directions.

#### Evolution characteristics of typhoon disaster states

**Figure 9:**
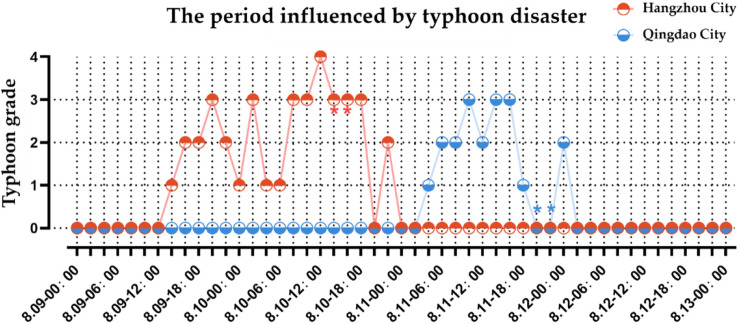
Different cities are influenced by typhoon disasters at different periods.

Adopting a temporal scale of 2 h, the extraction of typhoon disaster state information for Hangzhou city and Qingdao city is conducted, followed by the identification of the typhoon state type (Fig. 9). The time periods impacted by Typhoon Lekima in these two cities exhibit significant differences. Hangzhou city, being close to the typhoon’s landing point, experiences a prolonged impact as the typhoon moves slowly with high wind strength. On the other hand, when Typhoon Lekima affects Qingdao city, it is already in the late stages of its life cycle, resulting in a substantial weakening of the typhoon’s wind strength. Consequently, the impact on Qingdao city is shorter, and the wind strength is lower compared to Hangzhou city.

#### Application prospects

The method proposed in this paper provides robust support for enhancing the service capability of textual big data resources in disaster management. TDSIE, from the perspectives of static composition and dynamic process, constructs the basic framework for acquiring and reconstructing typhoon disaster information, thereby offering methodological insights for the extraction of other types of persistent disaster information. Extracting disaster state information from multiple sources of textual data enhances the comprehensiveness of disaster detection, thereby supporting various aspects of disaster management such as warning, monitoring, command, and assessment. In TDSIE, spatio-temporal features serve as crucial foundations for element identification and information fusion. However, this method overlooks the vagueness inherent in time and space information, thus it cannot handle metaphorical time expressions (e.g. when the house collapses) or spatial relationships that require reasoning (e.g. opposite to the building). The natural language expression in textual data often exhibit vagueness, and the existing TDSIE method are not suitable for social media texts with obvious colloquialism.

## Conclusion

The states represent segments in the development process of typhoon disasters. Extracting state information of typhoon disasters is a prerequisite for exploring their dynamic characteristics and analyzing evolutionary patterns. Due to the scarcity of annotated corpora specifically for typhoon disaster states, prevalent methods such as pattern matching and machine learning for event extraction cannot be directly applied. The research conclusions encompass two aspects: (1) This study comprehensively analyzes the features of spatio-temporal information elements, spatio-temporal semantic units, and spatio-temporal clues contained within the text. By integrating spatio-temporal features with methods such as part-of-speech tagging, state element identification, and state information fusion, it achieves the information extraction of typhoon disaster states from Chinese texts. The TDSIE addresses issues faced by generic disaster information extraction methods, such as dispersed disaster information and diverse spatio-temporal granularities. (2) Taking Typhoon Lekima as a case study, the analysis of extracted typhoon disaster state information from perspectives such as spatial patterns, temporal relationships, and evolutionary trends can provide a holistic reference for the timely detection of the dynamic processes of typhoon disasters.

In future research, the precision of information extraction of typhoon disaster states will be enhanced by expanding the core set of state elements, optimizing the effectiveness of semantic analysis in coreference resolution, and improving the judgment of timeliness. Additionally, studying reasoning methods for vagueness spatio-temporal information in typhoon states enhances the applicability of TDSIE to other social media textual data.

## Data Availability

The datasets analysed during the current study are not publicly available due to private proprietary but are available from the corresponding author on reasonable request.
